# Baicalin Downregulates RLRs Signaling Pathway to Control Influenza A Virus Infection and Improve the Prognosis

**DOI:** 10.1155/2018/4923062

**Published:** 2018-02-26

**Authors:** Peng Pang, Ke Zheng, Sizhi Wu, Huachong Xu, Li Deng, Yucong Shi, Xiaoyin Chen

**Affiliations:** ^1^College of Traditional Chinese Medicine, Jinan University, Guangzhou, Guangdong 510632, China; ^2^Department of Disease Control and Prevention, No. 371 Central Hospital of the People's Liberation Army, Xinxiang, Henan 453000, China

## Abstract

The objective of this study is to investigate the effects of baicalin on controlling the pulmonary infection and improving the prognosis in influenza A virus (IAV) infection. PCR and western blot were used to measure the changes of some key factors in RLRs signaling pathway. MSD electrochemiluminescence was used to measure the expression of pulmonary inflammatory cytokines including IFN-*γ*, TNF-*α*, IL-1*β*, IL-2, IL-4, IL-5, IL-6, IL-10, IL-12p70, and KC/GRO. Flow cytometry was used to detect the proportion of Th1, Th2, Th17, and Treg. The results showed that IAV infection led to low body weight and high viral load and high expression of RIG-I, IRF3, IRF7, and NF-*κ*B mRNA, as well as RIG-I and NF-*κ*B p65 protein. However, baicalin reduced the rate of body weight loss, inhibited virus replication, and downregulated the key factors of the RLRs signaling pathway. Besides, baicalin reduced the high expression inflammatory cytokines in lung and decreased the ratios of Th1/Th2 and Th17/Treg to arouse a brief but not overviolent inflammatory response. Therefore, baicalin activated a balanced host inflammatory response to limit immunopathologic injury, which was helpful to the improvement of clinical and survival outcomes.

## 1. Introduction

Influenza is an acute respiratory disease caused by the influenza virus, which threatens public health. The unforgettable 1918 pandemic Spanish influenza infected over one billion people and caused approximately 50 million deaths throughout the world [1]. Over the past two decades, influenza viruses, such as H1N1, H5N1, and H7N9, took turns to attack human health, which contributed substantially to the global health burden.

Currently, the two most widely used antiviral drugs are adamantanes and neuraminidase inhibitors (NAIs). They have obtained some positive anti-influenza treatment results via inhibiting the process of virus replication. However, the transmission of drug-resistant virus strains [2–4] limits the curative effects in dealing with the future influenza pandemic. Besides, the positive control drug ribavirin used in this study also shows a good antiviral effect. As a vRNA lethal mutagen resembling guanosine or adenosine, ribavirin causes mutations in RNA replication [5], which stops viral protein synthesis and limits the vRNA replication [6]. However, highly effective dose and adverse reactions limit ribavirin using as an anti-influenza drug. As Chinese herbal medicines have played admirable roles in anti-influenza [7, 8], they may have an excellent development in the future.

**Figure 1:**
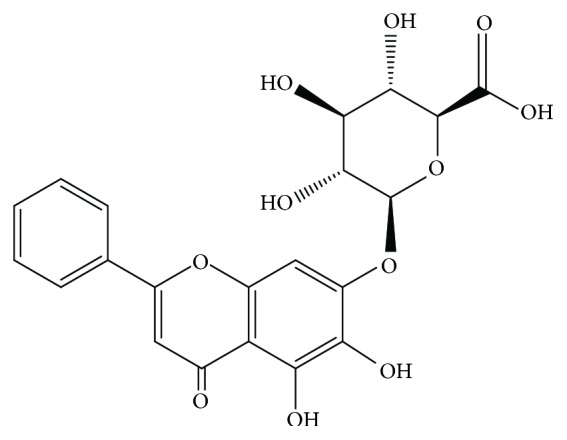
Chemical structure of baicalin.

*Scutellaria baicalensis* Georgi is a commonly used herbal medicine, which exhibits a variety of therapeutic effects in traditional formulations. Baicalin (Figure 1), as a main effective component of* Scutellaria baicalensis* Georgi, is a flavonoid compound isolated from the root of the herbal medicine [9]. Previous studies have shown that baicalin had clear inhibitory effects against neuraminidases of H1N1 and H3N2 viruses [10], and it modulated the function of the IAV-encoded NS1 protein [11]. Currently, most studies about anti-influenza effect of baicalin focused on the mechanism of virus replication inhibition. However, its good anti-influenza effect also represents the protective effect in controlling the pulmonary infection induced by influenza. How baicalin affects the innate immune pathway and limits the immunopathologic injury of influenza-induced pulmonary infection has not been fully explained. Therefore, this study focused on the protective effect of baicalin in influenza infection, especially the effect on RLRs innate immune pathways and the improvement of prognosis.

## 2. Materials and Methods

### 2.1. Reagent

The anti-CD4-FITC (#11-0041-81), anti-IFN-*γ*-APC (#17-7311-81), anti-IL-4-PE (#12-7041-81), anti-IL-17-PE-Cyanine7 (#25-7177-80), anti-CD25-APC (#17-0251-81), and anti-Foxp3-PE (#12-5773-80) were produced by eBioscience, San Diego, CA, USA. The RIG-I (CST#3743S) and NF-*κ*B p65 (CST#8242P) antibodies were produced by Cell Signaling Technology Inc., Danvers, MA, USA.

### 2.2. Animals

The sample size was calculated using the software PASS 11 (Kaysville, Utah, USA) under *α* = 0.01 and *β* = 0.1. Forty-eight specific pathogen-free C57BL/6j mice (half males and half females) weighing 20 ± 2 g were purchased from Laboratory Animal Center (Guangzhou University of Traditional Chinese Medicine, Guangzhou, China). Mice were kept in SPF laboratory animal room in a controlled environment (22°C ± 1°C and 50% ± 5% relative humidity), with free access to food and water for 7 days. The experiment was performed under the supervision and assessment of the Laboratory Animal Ethics Committee of Jinan University. Every experiment operation abided by the Statute on the Administration of Laboratory Animal approved by China Council 1988.

### 2.3. Grouping and Treatment

Forty-eight mice were divided randomly into 4 groups and each group had 12 mice with half males and half females. The mice were treated as follows: Group A: normal control group* (normal con)*; Group B: IAV control group* (V con)*; Group C: IAV + ribavirin treatment group (*V ribavirin*; 75 mg/kg); Group D: IAV + baicalin treatment group (*V baicalin*; 15 mg/kg).

Mouse-adapted strain of human influenza virus A/FM1/1/47 (H1N1) used in this experiment was provided by Department of Pathogenic Microbiology and Immunology, Jinan University. After fully anesthetized by inhalation of diethyl ether, mice in* V con*,* V ribavirin*, and* V baicalin* were infected by intranasal application of 80% LD50 influenza A virus suspension once (d1), while mice in* normal con* received saline as a blank control. From the second day, mice in* V ribavirin* and* V baicalin* received the drug treatment by intragastric gavage, respectively, for 5 days (d2–d6). All the mice were euthanized at the 7th day. Baicalin (7-D-glucuronic acid-5,6-dihydroxyflavone, purity > 95%) was purchased from Aladdin Company, Shanghai, China, and dissolved in polyethylene glycol (PEG, provided by Department of Chemistry, Jinan University, Guangzhou, China). As a kind of nontoxic solvent, PEG increased the solubility of baicalin [12], which also improved the bioavailability. Ribavirin (H51023508) was produced by Sichuan Baili Pharmaceutical Company, Sichuan, China.

### 2.4. Body Weight Changes and Lung Index

Weights of mice were taken daily. All mice were kept off food and water for 8 hours and then weighed before euthanized. The lungs were aseptically removed and weighed. The lung index was determined as an indication of lung inflammation and calculated according to the following formula: lung index = lung weight (g)/body weight (g) *∗* 100%.

### 2.5. Examination of Histopathology

The lung was separated after the animals were euthanized. About 5 mm *∗* 5 mm lung tissue was removed and fixed with 4% paraformaldehyde. The appropriate lung part was treated by rinsing, dehydration, treatment by a transparent agent, paraffin imbedding, and other steps. Paraffin-embedded tissue samples were sectioned, parched, dewaxed, hydrated, and then stained with hematoxylin and eosin. The central tissue organization was observed under a 200x microscope.

### 2.6. RT-qPCR of IAV Amplification and the Relative Expression of RIG-I, IRF3, IRF7, and NF-*κ*B mRNA

The lung was removed after the mice were sacrificed. The mRNA expression of RIG-I, IRF3, IRF7, and NF-*κ*B was measured using quantitative real-time reverse transcriptase PCR (RT-qPCR), as well as the influenza virus A/FM1/1/47 (H1N1) mRNA amplification. Total RNA was extracted by RNAiso Plus according to the instructions of the manufacturer. cDNA synthesis and real-time PCR were carried out using the CFX Connect Real-Time PCR Detection system (BIO-RAD, USA) with Prime Script RT reagent kits and SYBR Premix EX Taq II according to the manufacturer's instructions. Primers were synthesized by Generay Biotech Co. (Shanghai, China). Prime Script RT reagent kits (RR047A) and SYBR Premix EX Taq II (RR820A) were produced by TaKaRa, Japan. Primers for RT-qPCR are presented in Table 1.

After RT-qPCR, the 2^−ΔΔCT^ method was used for analysis of the relative gene expression levels. Each sample was measured three times and averaged to ensure accuracy. Gene expression in* V con*,* V ribavirin*, and* V baicalin* was expressed relative to the normal control* normal con*.

### 2.7. Western Blot for RIG-I and NF-*κ*B p65 Protein

Proteins were extracted from the lung tissues and then quantified using BCA protein assay kits. An equal amount of protein (20 *μ*g/lane) was fractionated using an electrophoresis system (BIO-RAD) on 10% polyacrylamide gels and then transferred to PVDF membranes. Membranes were, respectively, incubated with antibodies at an appropriate dilution of glyceraldehyde-3-phosphate dehydrogenase (GAPDH), RIG-I (1 : 1000), and NF-*κ*B p65 (1 : 1000) at 4°C overnight. Membranes were washed and incubated with secondary antibody for 1 h at room temperature. Protein bands were detected using an electrochemiluminescence (ECL) kit according to the manufacturer's instructions and photographed.

### 2.8. MSD for Inflammation Cytokines

Meso Scale Discovery (MSD) electrochemiluminescence was used to measure the cytokines that were important in inflammation response and immune system regulation. The supernatant of lung tissue was extracted to measure cytokines level in lung. V-PLEX Proinflammatory Panel 1 Mouse Kit (#K15048D-1, Meso Scale Discovery, Rockville, Maryland, USA) was purchased from Univ-bio Company, Shanghai, China. It provided an assay-specific component for the quantitative determination of IFN-*γ*, IL-1*β*, IL-2, IL-4, IL-5, IL-6, IL-10, IL-12p70, KC/GRO, and TNF-*α*. According to the manufacturer's instruction, reference standard was gradient dilution to make manufacture standard curve. Samples were diluted to the same concentration and added to Proinflammatory Panel 1 Plate, respectively. The samples were treated as in the following steps: incubation at room temperature, plate washing, addition of antibodies, incubation again, and addition of reading buffer, before detected and analyzed by MSD QuickPlex SQ120 (Meso Scale Discovery, Rockville, Maryland, USA).

### 2.9. Flow Cytometry for Evaluating T Cells Subsets

The spleen was isolated in a bioclean environment and ground. Splenocytes were isolated from the spleen by lymphocyte separation medium. After centrifugation and cell collection, splenocytes were washed and viable cells counted. Half of the splenocytes were stimulated with PMA/Ionomycin and BFA/Monensin for 4 h. The cells were washed and then fixed/permeabilized in the fixation/permeabilization and permeabilization buffers and stained with anti-CD4-FITC, anti-IFN-*γ*-APC, anti-IL-4-PE, and anti-IL-17-PE-Cyanine7. The other half splenocytes were fixed/permeabilized directly and stained with anti-CD4-FITC, anti-CD25-APC, and anti-Foxp3-PE. Flow cytometry was performed on a BD FACS verse flow cytometer (BD Biosciences, Franklin Lakes, NJ, USA) and analyzed using FlowJo (TreeStar, Ashland, Ore) analysis software.

### 2.10. Statistical Analysis

All experiments were performed at least three times and the results were from representative experiments. Statistical analyses were carried out using SPSS Statistics 20 (IBM Software, New York, USA) and drawn using GraphPad Prism 5 (GraphPad Software, San Diego, California, USA). All data are presented as Mean ± SD. Comparison among groups were done by using one-way ANOVA, followed by Student-Newman-Keuls tests. A *p* value of <0.01 was considered to be statistically significant.

## 3. Results

### 3.1. Changes of Body Weight and Lung Index

**Figure 2:**
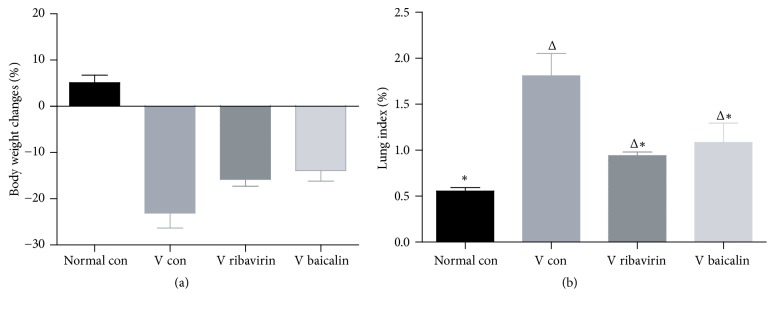
Changes of body weight and lung index. (a) Changes of body weight in 7 days. Body weight changes (%) = (BW_d1_ − BW_d7_)/BW_d1_  *∗* 100%; BW = body weight. (b) Changes of lung index. Lung index = lung weight/BW_d7_  *∗* 100%. ^Δ^*p* < 0.01 compared with* normal con. *^*∗*^*p* < 0.01 compared with* V con*.

Figure 2(a) shows the total changes of mice weight in the process of study. Influenza virus infection led to a significant decrease in mice weight, while ribavirin and baicalin treatment controlled the decline. Lung index was used to illustrate the severity of pulmonary inflammation. As the inflammatory exudation might increase the weight of lung, the greater lung index meant the more severe lung inflammation. According to Figure 2(b), the lung index of* V con* was statistically greater than* normal con*, which indicated inflammation response existent in mice lung. The lung index of* V ribavirin* and* V baicalin* showed a statistical downtrend compared to* V con*. That testified a slighter inflammatory reaction which occurred in* V ribavirin* and* V baicalin* than in* V con*.

### 3.2. Change of Lung Tissue

Pathological lung tissue is a more intuitionistic evidence of pulmonary inflammation. According to the scale in the following pictures, the view of the microscope is 200x.

**Figure 3:**
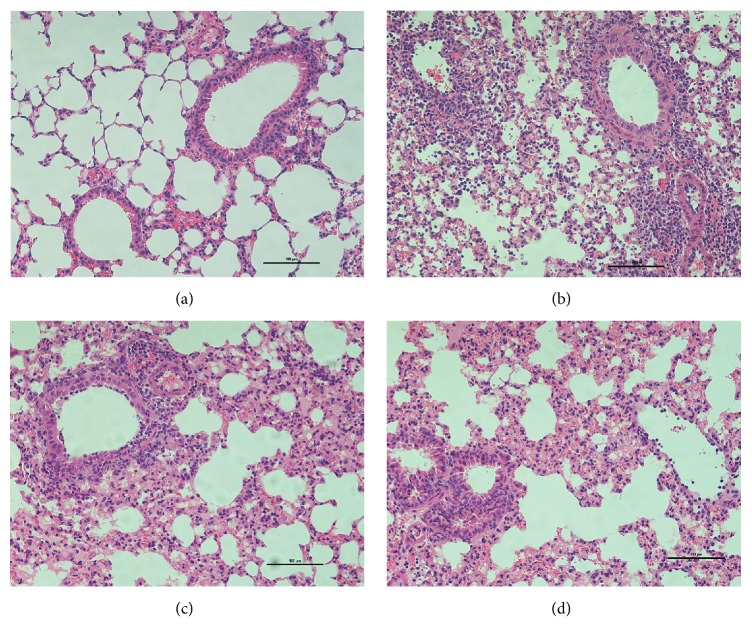
Effects of baicalin and ribavirin on the histological characterization in lung. (a) The normal condition of lung tissue. (b) Pathological damage after IAV infection. (c) Therapeutic effect of ribavirin. (d) Therapeutic effect of baicalin. All images obtained at ×200 magnification.

Figure 3 shows the change of histological characterization in lung. As the blank control,* normal con* showed the normal condition of lung tissue. Mice pulmonary alveoli showed structural integrity and regular shape that bronchial mucosal epithelium and muscularis mucosae were intact, without inflammatory cells infiltrating around (Figure 3(a)). In the IAV infection model* V con*, the bronchus, submucosal vessels, and the around lung tissue were infiltrated by a mass of lymphocytes infiltration (Figure 3(b)). The epithelia shed partly and part of the alveolar space atrophied resulting in compensatory dilatation of other alveolar cavities. Compared with* V con*, the inflammatory cells infiltration in lung tissue was obviously less and lighter in* V ribavirin* and* V baicalin*. The bronchial mucosa was relatively intact and less inflammatory cells assembled around the pulmonary alveoli (Figures 3(c) and 3(d)).

### 3.3. IAV Amplification in Mice Lung

To confirm influenza virus replication in the lung, the relative expression of the influenza A virus (IAV) genome was detected by quantitative reverse transcription PCR (RT-qPCR). The severity of influenza infection was associated with the amplification level of influenza virus.

**Figure 4:**
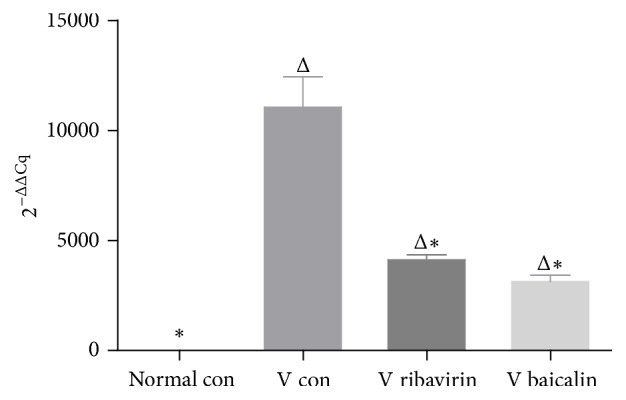
Effect of baicalin and ribavirin on changing the relative expression levels of the IAV replication. Total RNA was extracted from lung tissue of mice. ^Δ^*p* < 0.01 compared with* normal con*. ^*∗*^*p* < 0.01 compared with* V con*.

The relative expression level of IAV replication is displayed in Figure 4. The high expression level of IAV replication in* V con* certified that the influenza mice model was successfully established. The viral replication expression in* V ribavirin* and* V baicalin* was statistically significant decreased compared to* V con*. It illustrated that ribavirin and baicalin restrained IAV amplification in mice lung.

### 3.4. Relative mRNA Expression of RIG-I, IRF3, IRF7, and NF-*κ*B

Retinoic acid-inducing gene I-like receptors (RLRs) signaling pathway was well-known innate immune pathway, which can identify influenza viruses in the cytoplasm and regulate the immune response. The relative mRNA expression of RIG-I, IRF3, IRF7, and NF-*κ*B was measured to investigate how baicalin affected the important factors of RLRs signaling pathway.

**Figure 5:**
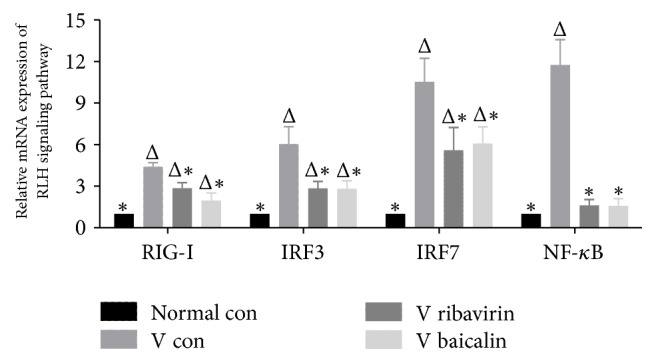
Relative mRNA expression of RIG-I, IRF3, IRF7, and NF-*κ*B in RLRs signaling pathway. ^Δ^*p* < 0.01 compared with* normal con*. ^*∗*^*p* < 0.01 compared with* V con*.

Figure 5 shows the relative mRNA expression of RIG-I, IRF3, IRF7, and NF-*κ*B. Compared to* normal con*, the relative mRNA expression of RIG-I, IRF3, IRF7, and NF-*κ*B was significantly increased in* V con*. The relative expression of RLRs signaling pathway mRNA in* V ribavirin* and* V baicalin* showed a marked decline with statistically significant differences compared with* V con*. The statistical results illustrated that ribavirin and baicalin downregulated the expression of RLRs signaling pathway at the genetic level.

### 3.5. Protein Expression of RIG-I and NF-*κ*B p65

RIG-I and NF-*κ*B in RLRs signaling pathway were further studied at the protein level to observe the effects of IAV infection and also the treatment results of ribavirin and baicalin.

**Figure 6:**
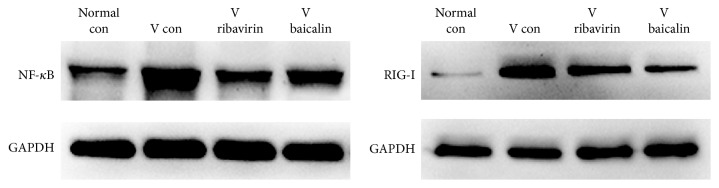
Relative protein expression of RIG-I and NF-*κ*B.

The relative expression of RIG-I and NF-*κ*B protein is showed in Figure 6. Compared to* normal con*, the expression of RIG-I and NF-*κ*B p65 was markedly increased in* V con*. The expression of RIG-I and NF-*κ*B p65 in both* V ribavirin* and* V baicalin* was decreased compared with* V con*. The investigation illustrated that baicalin downregulated protein expression of RIG-I and NF-*κ*B in RLRs signaling pathway.

### 3.6. Expression of Inflammatory Cytokines

MSD was used to detect the expression of inflammatory cytokines. Ten inflammation-related cytokines concentrations were measured to verify the intervention effect of baicalin. Thereinto, IFN-*γ*, IL-1*β*, IL-2, IL-5, IL-6, IL-12p70, TNF-*α*, and KC/GRO were focused on their proinflammatory roles, while IL-4 and IL-10 attracted more attention to their inflammation suppression effect.

**Figure 7:**
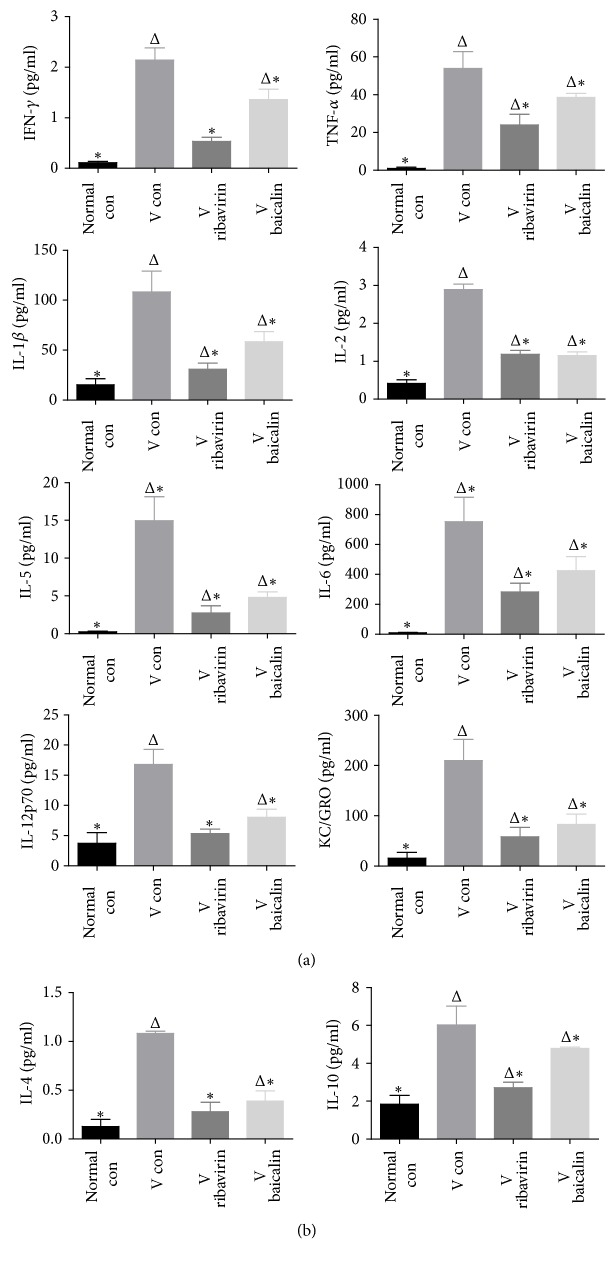
MSD was used to detect the expression of inflammatory cytokines. (a) The expression changes of proinflammatory cytokines including IFN-*γ*, IL-1*β*, IL-2, IL-5, IL-6, IL-12p70, TNF-*α*, and KC/GRO in lung. (b) The expression change of anti-inflammatory cytokines including IL-4 and IL-10 in lung. ^Δ^*p* < 0.01 compared with* normal con*. ^*∗*^*p* < 0.01 compared with* V con*.

The results of the ten inflammation cytokines' expression are displayed in Figure 7. On the general trend, the expression of cytokines, both proinflammatory and anti-inflammatory cytokines, showed a statistically significant increase in* V con* compared to* normal con*. The results indicated that IAV infection led to a persistent high expression of inflammatory cytokines, which was harmful to lung tissue. Compared with* V con*, all the ten inflammatory cytokines decreased in* V ribavirin* and* V baicalin* with statistical significance. It suggested that ribavirin and baicalin suppressed the occurrence of cytokine storm.

### 3.7. The Classification of CD4+ T Lymphocytes

CD4+ T lymphocyte differentiation was one of the inflammation characterizations. Flow cytometry was used to detect the proportions of the CD4+ T cell subsets Th1, Th2, Th17, and Treg. Furthermore, Th1/Th2 and Th17/Treg were used to represent the change of immunologic balance.

**Figure 8:**
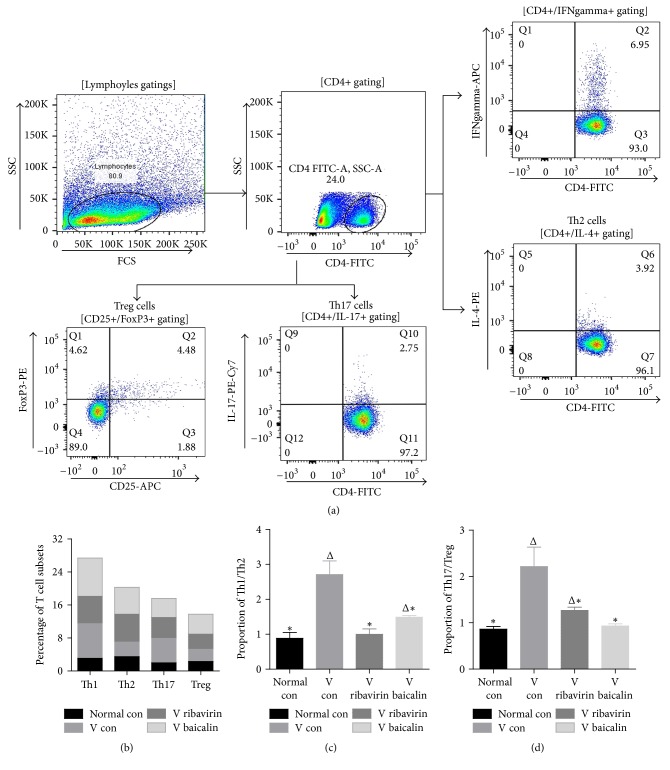
Flow cytometry was used to measure the proportion of the T cell subsets Th1/Th2 and Th17/Treg. (a) Flow cytometry analysis of CD4+ T cell subsets. (b) The percentage of T cell subsets including Th1, Th2, Th17, and Treg in different groups. (c) Changes in the proportion of Th1/Th2 cells. (d) Changes in the proportion of Th17/Treg cells. ^Δ^*p* < 0.01 compared with* normal con*. ^*∗*^*p* < 0.01 compared with* V con*.

According to Figure 8, the proportion of the CD4+ T cell subsets Th1 and Th17 appeared to be on the rise after infection, while Th2 and Treg were still similar to* normal con*. It led to an increase in proportions of Th1/Th2 and Th17/Treg in* V con*. In* V ribavirin and V baicalin*, Th2 and Th1 raised synchronously, which resulted in lower proportions of Th1/Th2. Th17 and Treg showed a similar trend in* V ribavirin and V baicalin*. Furthermore, ribavirin showed a better inhibitory effect on Th1/Th2 differentiation, while baicalin performed better on restraining the Th17/Treg differentiation.

## 4. Discussion

Host antiviral immune responses are crucial in the host's process to eliminate virus. Pathogen-associated molecular patterns (PAMPs) are detected by the “sensor” that is known as pattern recognition receptors (PRRs), which play a crucial role in triggering host innate immunity. Recent studies have identified several innate immune receptors including Toll-like receptors (TLRs), nucleotide-binding oligomerization domain-like receptors (NLRs), and RIG-I-like receptors (RLRs) [13]. Here we focused on the effect of baicalin on the RLRs signaling pathway in innate antiviral immunity, which was essential for the control of infection by RNA viruses. The retinoic acid-induced protein I (RIG-I) was the pivotal cytoplasmic pathogen recognition receptors in RLRs signaling pathway [14]. Activated RIG-I signal interacted with the adapter protein MAVS and led to a signaling cascade to activate the transcription factors IRF3, IRF7, and NF-*κ*B [15]. Influenza A virus activated RIG-I protein and led to an activation of downstream factors IRF3, IRF7, and NF-*κ*B. RIG-I controlled IRF-dependent and NF-*κ*B-dependent cytokine synthesis and activated inflammatory in response to influenza viruses [16]. Thereinto, the IFN regulatory factors IRF3 and IRF7 were the crucial regulator of type I interferons against pathogenic infections [17]. Type I interferon (IFN*αβ*) was widely recognized to have the antiviral function, which induced abundant proteins that impair viral replication in infected cells [18]. NF-*κ*B was generally considered to be the key transcription factor that affects the secretion of type I interferon and other inflammatory cytokines, such as IL-1 and TNF [19]. In addition, IFN-*γ* potentiated CXCL10 expression via the NF-*κ*B-related pathway against viral infection [20].

However, host antiviral immune responses play as a double-edged sword in pathogenesis and prognosis of acute lung injury caused by influenza. A robust host innate immune response, which contributes to viral clearance, can worsen the severity of lung injury. Hyperactive RLRs signaling pathway might mediate pathological damage by persistently activating the inflammatory cytokines [21]. For instance, excessive IFN*αβ* contributed to disease, by leading to high levels of inflammatory cytokines and chemoattractant cytokines, which recruited massive inflammatory cells and caused acute immunopathology injury [22, 23]. Hence immune responses to infection required a fine balance between viral clearance and host preservation. According to the gene and protein detection, baicalin downregulated some key factors of RLRs signaling in IAV infection. By comparison, NF-*κ*B mRNA showed a greater degree of downregulation than RIG-I. The reason might be that there were multiple innate immune pathways that acted on the NF-*κ*B protein at the same time. RIG-I was upstream connector protein of RLRs signaling pathway, while NF-*κ*B was downstream effector protein of both RLRs and TLRs. A study indicated that baicalin also downregulated the TLR7/MyD88 signaling pathway of H1N1 infected mice [24]. Therefore, baicalin downregulated some key factors of RLRs signaling pathway to inhibit hyperactive innate immune response in IAV infection.

The inflammatory response is known to be a clear characteristic of the host's process to eliminate virus. However, as much of immune pathological damage that occurs during severe infection can be attributed to this very same mechanism [25], the intensity of inflammatory response may be closely related to the prognosis of the disease. When inflammation fails to keep the balance between pathogen clearance and prevention of host-tissue damage, an uncoordinated immune response may lead to devastating diseases and even death [26]. For instance, the severe disease of 2009 H1N1 pandemic influenza in humans was characterized by the presence of hypercytokinemia, which was associated with uncontrolled influenza virus replication [27]. Meanwhile, it was reported that the recovered and discharged patients had significantly lower cytokine storm profiles than the dead ones [28]. Thus, as a principal immunopathological mechanism, the cytokine storm had a close relationship with poor clinical outcome and pathogenesis in aberrant pulmonary immune responses of influenza virus infection [29]. According to the MSD results, the proinflammatory cytokines were of high expression after IAV infection. For instance, IFN-*γ*, TNF-*α*, IL-1*β*, IL-2, IL-5, IL-6, and IL-12p70 were highly upregulated to exert their inflammatory activity, as well as the chemotactic factor GRO/KC (CXCL1). Although the proinflammatory cytokines were important in controlling extrapulmonary viral spread [30–35], they also contributed to exaggerating immune response in lung inflammation [36]. The anti-inflammatory cytokines, IL-4 [37] and IL-10 [38], expressions also increased for the influenza virus infection. Obviously, after influenza virus infection, a variety of inflammation-related cytokines were produced and released in large quantities, which might lead to “cytokine storm” and result in immune injury in lung consequently.

Our study showed that the expressions of the inflammatory cytokines were signally downregulated after baicalin treatment. The balance between proinflammatory cytokines and anti-inflammatory cytokines during the progression of influenza infection determined the outcome of immune-mediated injury in influenza infection. Although expressions of cytokines were reduced, they were sufficient to terminate the infection. Our study testified that baicalin prevented the occurrence of cytokine storm by downregulating the expression of inflammatory-related cytokines. Baicalin helped the cytokines arouse a brief but not overviolent inflammatory response. It suppressed the overactivation of the inflammatory response and prevented exacerbation of immune-mediated injury.

The cytokine expressions and pathologic pictures of lung explained the changes of local inflammatory conditions, and the differentiation of splenocytes displayed the systemic effects of virus infection and treatment. Therefore, to systematically expound how baicalin therapy activated relatively a balanced innate inflammatory response, we assessed the effects of baicalin on CD4+ T cells, such as Th1, Th2, Th17, and Treg. Similar to cytokines, T lymphocytes subsets exerted different functions in inflammatory reaction [39]. Th1 and Th17 played a role in promoting inflammation against pathogens [40, 41], while Th2 and Treg had effects on suppressing inflammation [42, 43]. According to the flow cytometry results, IAV increased the proportion of Th1 and Th17, while Th2 and Treg were still similar to* normal con*. It led to a rise in the proportions of Th1/Th2 and Th17/Treg and suggested that IAV promoted Th1 and Th17 types inflammation response. It was critical to keep the balance of pro- and anti-inflammatory mechanisms for maintaining immune homeostasis. The statistics showed that baicalin declined the ratio of Th1/Th2 and Th17/Treg by increasing the proportions of Th2 and Treg. It indicated that baicalin attenuated the influenza-induced Th1 and Th17 types inflammatory reaction. It was coincident with the change of cytokines. Furthermore, baicalin showed a weaker inhibition of Th1/Th2 than Th17/Treg, which implied that baicalin might run a mild type 1 inflammatory response to anti-influenza. If the immunoreaction was enough to clear the infection and not cause tissue damage, it might be an optimal response to influenza viral infection. Consequently, baicalin impacted the differentiation tendency of CD4+ T cells and run a mild type 1 inflammatory response to anti-influenza, which controlled inflammatory damage caused by IAV infection.

## 5. Conclusions

In conclusion, baicalin downregulated some key factors of RLRs signaling pathway and activated a balanced host inflammatory response, which was helpful to the improvement of clinical and survival outcomes. Baicalin helped the cytokines arouse a brief but not overviolent inflammatory response to limit local immunopathologic injury. Baicalin also showed a protective systemic effect in IAV influenza infection, as it restrained the inflammatory differentiation of CD4+ T cells and run a mild type 1 inflammatory response.

## Figures and Tables

**Table 1:** Primers used for RT-qPCR analysis.

Gene	Primer	Sequence
IAV	Forward primer	5′-GACCAATCCTGTCACCTCTGAC-3′
	Reverse primer	5′-GGGCATTTGGACAAACGTCTACG-3′
RIG-I	Forward primer	5′- CAGAGCCAGCGGAGATAAC-3′
	Reverse primer	5′- GGTCAGGAGGAAGCACTTG-3′
IRF3	Forward primer	5′-TTGTGATGGTCAAGGTTGTTC-3′
	Reverse primer	5′-GGAGATAGGCTGGCTGTTG-3′
IRF7	Forward primer	5′-GCGTACCCTGGAAGCATTTC-3′
	Reverse primer	5′-GCACAGCGGAAGTTGGTCT-3′
NF-*κ*B	Forward primer	5′-ATTCTGACCTTGCCTATCTAC-3′
	Reverse primer	5′-TCCAGTCTCCGAGTGAAG-3′
GAPDH	Forward primer	5′-TGATGACATCAAGAAGGTGGTGAAG-3′
	Reverse primer	5′-TCCTTGGAGGCCATGTAGGCCAT-3′
